# Improving control over euthanasia of persons with psychiatric illness: Lessons from the first Belgian criminal court case concerning euthanasia

**DOI:** 10.3389/fpsyt.2022.933748

**Published:** 2022-07-19

**Authors:** Marc De Hert, Sien Loos, Sigrid Sterckx, Erik Thys, Kristof Van Assche

**Affiliations:** ^1^University Psychiatric Centre, Katholieke Universiteit Leuven, Leuven, Belgium; ^2^Department of Neurosciences, Centre for Clinical Psychiatry, Katholieke Universiteit Leuven, Leuven, Belgium; ^3^Leuven Brain Institute, Katholieke Universiteit Leuven, Leuven, Belgium; ^4^Antwerp Health Law and Ethics Chair, University of Antwerp, Antwerp, Belgium; ^5^Research Group Personal Rights and Property Rights, Faculty of Law, University of Antwerp, Antwerp, Belgium; ^6^Leuven Institute for Healthcare Policy, Katholieke Universiteit Leuven, Leuven, Belgium; ^7^Bioethics Institute Ghent, Department of Philosophy and Moral Sciences, Ghent University, Ghent, Belgium

**Keywords:** euthanasia, MAID, psychiatric disorders, end of life, legal, ethics, borderline personality, autism spectrum disorder (ASD)

## Abstract

**Background:**

Belgium is one of very few countries that legally allow euthanasia for suffering caused by psychiatric illness. In the first criminal trial in Belgium of physicians involved in euthanasia, three physicians recently faced the accusation of “murder by poisoning,” for allegedly having failed to comply with several requirements of the Belgian Euthanasia Law in granting the euthanasia request a woman suffering from psychiatric illness. Although all three physicians were acquitted, the case generated much debate among policy makers, medical professionals, and the general public.

**Method:**

We use this trial as the starting point for a critical analysis of the adequacy of the three-level control system established in the Euthanasia Law, as it is applied in the evaluation of euthanasia requests from persons who suffer unbearably from a psychiatric illness. This analysis is based on information presented during the criminal trial as well as information on the euthanasia that was published in the press.

**Results:**

Our analysis highlights substantial problems in the assessment and granting of the euthanasia request. The patient was euthanized without it having been substantiated that her psychiatric illness had no prospect of improvement and that her suffering could not be alleviated. The three-step control system enshrined in the Law and promoted by the Federal Control and Evaluation Commission for Euthanasia appears to have failed at each level.

**Conclusion:**

To evaluate requests for euthanasia for mental suffering caused by psychiatric illness, the requirements of the Belgian Euthanasia Law should be complemented by mandating the advice of two psychiatrists, and face-to-face discussions between all physicians involved. In parallel with the process of evaluating the euthanasia request, a treatment track should be guaranteed where reasonable evidence-based treatments and recovery-oriented options are tried.

## Introduction

**FIGURE 1:**
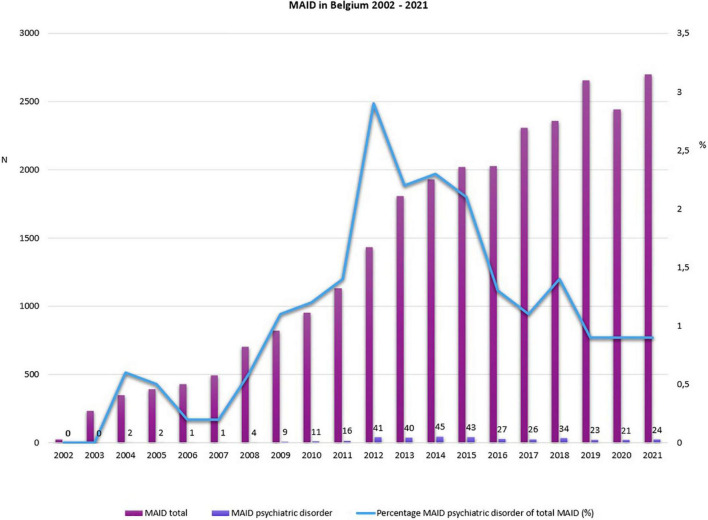
Medical assistance in dying (MAID) in Belgium (adapted from 6).

**FIGURE 2:**
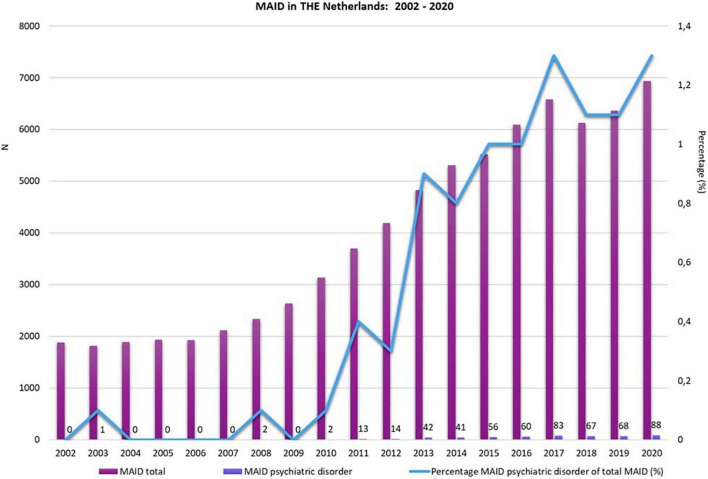
Medical assistance in dying (MAID) in The Netherlands see text footnote 1.

Medical assistance in dying (MAID) for unbearable mental suffering caused by a psychiatric illness is currently allowed in four countries. More specifically, non-terminally ill persons who suffer unbearably from a psychiatric illness can, subject to the fulfillment of stringent conditions, legally receive euthanasia in Belgium, physician-assisted suicide and euthanasia in The Netherlands and Luxembourg, and physician-assisted suicide in Switzerland. Whereas no cases have yet been reported in Luxembourg, the number of patients who request or receive MAID on the basis of a psychiatric illness is gradually increasing in Belgium, The Netherlands, and Switzerland (1–5). The most recent data for Belgium and The Netherlands are presented in Figures 1, 2. In recent years, the percentage of MAID in patients with a psychiatric disorder has remained stable between 1 and 2% of the total number of MAID cases. The most frequent primary diagnosis are mood disorders and personality disorders^1^. For Switzerland detailed longitudinal information on PAS and (bi)annual official reports are missing. Between 2010 and 2018 absolute numbers increased threefold (in 2018 1.8% of all deaths) (2). A retrospective study confirms the increase of PAS over time both in Swiss residents and people from other countries (6). Overall 13.1% of PAS was performed in people with psychiatric disorders (respectively 17.6% PAS in residents and 7.7% in foreigners). The main diagnosis was depression.

**FIGURE 3:**
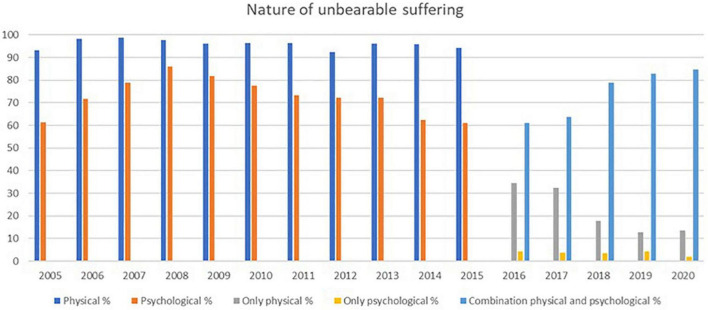
Nature of unbearable suffering (from the official reports of the FCECE)*. *Category of labels changed between 2015 and 2016.

In Belgium, the legal requirements for Euthanasia are set out in the Law on Euthanasia, adopted in 2002. A person who suffers from psychiatric illness can receive euthanasia for mental suffering if five cumulative conditions are fulfilled. First, the person should make a written euthanasia request him- or herself, and needs to be a legally competent adult. Second, the request should be voluntary, without external pressure, well-considered, and repeated. Third, the person should be in a medical condition without prospect of improvement. Fourth, the mental suffering has to be constant, unbearable, and cannot be alleviated. Fifth, the mental suffering should result from a serious and incurable psychiatric illness (7–10).

The Law describes in detail the procedure to be followed to guarantee that on a non-terminally ill person who suffers from a psychiatric illness no euthanasia is performed in breach of these conditions. In this regard, the Federal Control and Evaluation Commission for Euthanasia (FCECE)^2^ has emphasized that the Law prescribes three levels of control. The first level of control is the “auto control” by the individual physician who is confronted with the euthanasia request and agrees to perform euthanasia when the five legal requirements would be met. To assess the fulfillment of these criteria, that physician—termed “the attending physician” in the Law—needs to review the patient’s medical file, to examine the patient, and to have several conversations with the patient spread out over a reasonable period of time.

The second level of control is the “peer control,” involving two consulted physicians who also have to assess the fulfillment of some of the criteria, and in this way control whether the attending physician is acting in accordance with the Law. More specifically, the attending physician must consult a second physician (i.e., the first consulted physician) to ascertain the patient’s constant and unbearable mental suffering that cannot be alleviated, and the serious and incurable nature of the psychiatric illness that is causing the suffering. In addition, the attending physician must also consult a third physician (i.e., the second consulted physician), who needs to be a psychiatrist,^3^ and who must ascertain the voluntary, well-considered, and repeated nature of the euthanasia request, and the patient’s constant and unbearable mental suffering that cannot be alleviated. If, after having consulted the two other physicians, the attending physician is convinced that the euthanasia request fulfills all five the legal criteria, the euthanasia can be performed. As in other cases of non-terminal illness, a euthanasia on a non-terminally ill person who suffers from a psychiatric illness can only be performed after at least 1 month has passed counted from the date of the patient’s first request. Importantly, to guarantee that the consulted physicians can each come to an autonomous conclusion, the Law requires them to be independent vis-aÌ-vis the patient as well as vis-aÌ-vis the attending physician. The Law does not specify how “independence” should be understood, but in its information brochure for physicians, the FCECE has emphasized that the consulted physicians should not have a family tie or a hierarchical relation with the attending physician and no regular therapeutic relationship with the patient. In its latest report, the FCECE clarified that “independence” should not be construed as meaning that the consulting physician must never have met the patient nor know his or her medical history.

If the euthanasia is performed, a third level of control is initiated, involving an *a posteriori* review of the euthanasia by the FCECE. The Law stipulates that the attending physician needs to submit a registration form to the FCECE within four working days after the euthanasia has been performed. The FCECE examines the registration form to determine whether the legal criteria were met. If it decides with a two-thirds majority vote that the euthanasia had been performed in violation of the Law, it refers the case to the Public Prosecutor. Importantly, the Law itself does not specify the crime committed if the legal criteria have not been met. As a result, one has to resort to the classification of crimes listed in the Criminal Code, meaning that a violation of the Law will amount to the crime of “murder by means of poisoning.” Whether the person who was euthanized had consented would in this case not be material, since under the Criminal Code the consent of a “victim” cannot be a ground for justification of a murder charge (4, 8, 11).

In January 2020, for the first time in the history of Belgian euthanasia practice, an attending physician and two consulted physicians involved in a euthanasia case appeared before court for “murder by means of poisoning.” The trial was held before the Court of Assize in Ghent and concerned the euthanasia for mental suffering caused by a psychiatric illness of a patient in her late 30s, performed in 2010. The jury members had to decide whether in euthanizing her the physicians had violated the conditions and procedure set out in the Euthanasia Law and, consequently, would have committed murder. Since euthanasia on psychiatric patients remains a highly controversial topic, the criminal trial resulted in a media storm and heated discussions among policymakers, medical professionals, and the general public. In the month before and after the trial 1,215 news items regarding the trial were published in Belgian newspapers and online news platforms^4^.

## Method

We take the euthanasia of this patient as the starting point for a critical analysis of the adequacy of the three-level control system established in the Euthanasia Law, as it is applied in the evaluation of euthanasia requests from persons who suffer unbearably from a psychiatric illness. In this way, we will at the same time examine whether in this particular case the eligibility criteria had been fulfilled. Possible ways to improve the three-level control system will also be discussed.

Our analysis of the euthanasia of the patient is based on information presented during the criminal trial (attended by MDH, SS, and KVA) as well as on information published in the press. ET was appointed as a psychiatrist by the family shortly after the euthanasia to enable them to access the medical case-notes. Although such access is a right of family members after the death of a relative, it was nonetheless denied.

In order to maximize privacy and confidentiality, we only mention personal data regarding the parties involved when necessary for our analysis.

## Background to the euthanasia and the criminal trial

The patient was diagnosed with borderline personality disorder (BPD) when she was 19. Her course of BPD was particularly turbulent, with episodes of suicidal behavior, for which she was admitted to hospital more than 10 times, often for a longer period. Since she was last admitted to hospital at age 34, she received supportive counseling from a psychologist in the local community mental health center. In the final years before the euthanasia, she obtained a relative functional stability: she completed a graduate education, worked part-time, lived independently, and in 2009 moved in with a partner. However, according to some testimonies given at the trial, her private life was still tumultuous, characterized by occasional drug use and partner violence, and her job eventually proved to be very demanding. Mid-2009 her relationship ended, and her dreams of marriage and a family shattered. These major setbacks in her private and professional life confronted her with persistent and severe difficulties in coping with life and maintaining relationships.

At that time, the patient became severely depressed and suicidal, and she enquired with her psychologist and her general practitioner (GP) about the possibility of euthanasia. She was referred to another GP, who was officially registered by the largest Flemish right-to-die society as a physician willing to perform euthanasia, and who had followed several sessions of their professional training program on euthanasia. On 22 December 2009, she submitted her official, written euthanasia request to him. The GP agreed to act as attending physician and to evaluate the request. She herself had already contacted the two other physicians who would become involved in the euthanasia. The person who would officially act as the first consulted physician, but who would later maintain that he had been deceived, was her regular GP. The second consulted physician was a psychiatrist nationally known for her advocacy for the possibility of, and her expertise in, euthanasia for unbearable mental suffering in persons with a psychiatric illness. She had established an organization specialized in awareness raising about, and evaluating requests for euthanasia. That psychiatrist agreed to act as a consulted physician in evaluating a possible euthanasia request. During the evaluation of the request by the psychiatrist, the patient was referred for additional diagnostic testing, resulting in a “new” diagnosis of Asperger’s Syndrome (AS). Based on the advice of the psychiatrist, the attending physician granted the euthanasia request, referring to constant and unbearable mental suffering on the part of the patient, without possibility of alleviation, and resulting from BPD and AS. The euthanasia was performed on 27 April 2010.

The patient’s family was involved in one aspect of the evaluation process, by way of a conversation with the psychiatrist who acted as the second consulting physician. The family acknowledged her severe mental problems in the past, but found the problems at the time of the request very difficult to reconcile with granting the request and performing the euthanasia. They primarily questioned the new diagnosis and the fact that no new treatment had been proposed, let alone started. In their quest for answers, they re-contacted the different physicians and the FCECE after the euthanasia, and were confronted with conflicting statements by the physicians. They also discovered that at least some of the procedural requirements had not been fulfilled (e.g., the euthanasia registration document had not been sent to the FCECE in time), and that an additional psychiatrist that they had contacted to look into the patient’s medical file and euthanasia evaluation reports was eventually denied access.

After a formal complaint by one of the patient’s sisters, the Council Chamber, responsible for overseeing the judicial investigation and deciding whether sufficient indications of guilt exist to bring criminal charges, decided on 20 December 2016 that there were no grounds to refer the case to the trial court. This decision was mainly based on the fact that the FCECE had allegedly unanimously approved the case (although no minutes of the FCECE meeting in question were available). By contrast, upon appeal by the family, on 22 November 2018 the Chamber of Indictment found that there *were* sufficient grounds for referring the physicians, now accused of “murder by poisoning,” to the trial court (12). The decision by the Chamber of Indictment was based on a report from two experts appointed by the investigating magistrate, which concluded that not all requirements of the Euthanasia Law had been met.

The case was decided before the Court of Assize in Ghent, in a trial lasting from 17 to 31 January 2020. The jury members had the difficult task of judging the actions of the three physicians who had been involved in evaluating the nature, severity, and prospects of the patient’s complex psychiatric history and mental suffering in the context of a euthanasia request. In the trial a central role was given to new legal and medical experts appointed by the President of the Court of Assize. After an 8-hour deliberation in the final night, all three of the physicians were acquitted (13). The jury decided that the guilt of the attending physician, who had performed the euthanasia, had not been proven beyond a reasonable doubt. The first consulted physician was considered to have been misled by the attending physician in that he had not been made fully aware that the note he had reluctantly written in support of the patient’s euthanasia request would be used as one of the three legally required official advices. Finally, the jury found no elements that would indicate that the second physician, the psychiatrist, had not fulfilled the requirements of the Euthanasia Law that were relevant to her specific role in assessing the patient’s request.

However, the family successfully appealed to the Court of Cassation against the acquittal of the attending physician, citing a violation of an essential procedural requirement, namely the obligation of the jury to sufficiently motivate their decision. Since under Belgian law an acquittal by a Court of Assize remains standing even if quashed by the Court of Cassation, the case against the attending physician was referred to a civil court for a new assessment of the facts. If, contrary to the decision of the Assize jury, this court would find that the attending physician still had violated the criteria or procedure set out in the Euthanasia Law, this would result in awarding compensation for damages to the family. Currently, the civil court case is put on hold, since the judge has referred to the Constitutional Court preliminary legal questions on the interpretation of certain provisions in the Euthanasia Law and the legal implications of their violation (14).

Information presented at the trial before the Court of Assize made clear that in the euthanasia of the patient several legal requirements had not been met, and that the three-level control system established in the Euthanasia Law had in this case not functioned properly. We will now examine this more in detail.

## Auto control

The attending physician was required to evaluate the fulfillment of all five criteria specified in the Law. To assess the eligibility of the patient’s euthanasia request, he saw her three times and, according to his testimony before court, also had several conversations over the phone. The medical file that he kept on the assessment of the euthanasia request was virtually non-existent, as it contained only three short entries. It is clear that he personally only managed to ascertain that she was legally competent to make the request, and that her request was voluntary, well-considered, and repeated.

As to the condition of legal competence, it should be noted that assessing the decisional capacity of psychiatric patients who request euthanasia is a complex issue. Opponents of MAID in patients who suffer from a psychiatric illness list as one of their major concerns that a psychiatric illness can severely negatively impact upon a person’s competence. However, we see no reason to presuppose that a person suffering from a psychiatric illness would lack the required decisional capacity. Capacity should be assessed on a case-by-case basis, depending on the context and the nature and consequences of the decision (15–18). Although a high standard will thus need to be applied for decisions that concern life and death (19–21), a possible impression on the part of the medical professional or other persons that the decision from a patient who suffers from a psychiatric illness is irrational or unwise, should not be a decisive element. Instead, the determination of the patient’s decisional capacity should solely be established in reference to the quality of the decision-making process and the underlying values expressed. Importantly, capacity is task-specific and may vary over time (17, 18, 22–25).

Before the Court of Assize some concern was raised about whether the substance use and depression of the patient would have rendered her incompetent to request and receive euthanasia. However, the attending physician and all other healthcare professionals who had spoken to her in the months prior to her death testified that in their view there had been no doubt that she had the required decisional capacity and that her request was voluntary and well-considered.

There were similarly no doubts that the patient had repeatedly requested euthanasia spread over a reasonable period of time. She provided the attending physician with a written request on 22 December 2009, and again on 27 April 2010, moments before she was euthanized. That her wish was sincere and frequently repeated was also emphasized by the two consulted physicians, and many other healthcare professionals, family members, and friends who testified before court. To affirm her death wish the patient at the end of December 2009 also registered the two binding advance directives regarding end-of-life care that are legally allowed in Belgium. In the first one, she abstained from resuscitation and life-prolonging treatment, and in the other one, she expressed her wish to be euthanized in case of irreversible coma.

Whereas the attending physician had ascertained the fulfillment of the first two legal requirements, he lacked the competence to diagnose her mental suffering and complex psychiatric illness. As a result, he “out-sourced” the assessment of the three other requirements to the second consulted physician, the psychiatrist. At the trial, it became clear that the attending physician had not independently tried to verify whether the patient’s unbearable suffering could not be alleviated and resulted from a psychiatric illness without prospect of improvement—tasks that he was entrusted with under the Law but for which, as indicated, he as a GP didn’t have the expertise to properly perform. Rather, he had entirely relied on the opinion of the psychiatrist.

Moreover, despite having followed several sessions of a professional training program on euthanasia, the attending physician testified that, at the time of the euthanasia, he had limited knowledge of the Law. This was further evidenced by the fact that he had not been aware that the patient’s GP was legally not allowed to act as a consulted physician, in view of his lack of independence from her. On the day of the euthanasia, he even personally went to see the GP to obtain his legally required written “advice,” supporting the euthanasia that would take place the same evening. The attending physician also neglected to submit the registration form concerning the euthanasia to the FCECE within the legally specified four working days. It arrived there only 51 days later and contained several factual errors. Together with the registration form, he also sent the original files with the advices of the two consulted physicians—which should have better been kept in his medical file on the euthanasia of the patient.

## Peer control

As dictated by law, the peer control of the attending physician had to be performed by two physicians. Since the suffering that the patient requested euthanasia for was caused by a psychiatric illness, the second physician needed to be a psychiatrist.

### The first consulted physician

The first consulted physician was the patient’s GP, who was approached by the patient herself to issue a positive advice on her euthanasia request. She had been his patient for approximately 10 years, receiving active counseling and medication for her psychiatric problems. His role as her GP would have legally excluded him from acting as a consulted physician in her euthanasia request.^5^ However, at the trial, both he and the attending physician testified that they had been insufficiently familiar with this and other aspects of the Euthanasia Law.

Moreover, the GP lacked the expertise to verify the fulfillment of the two legal criteria—the patient’s constant and unbearable mental suffering that cannot be alleviated, and the serious and incurable nature of the psychiatric illness that is causing the suffering—whose separate, independent evaluation the Law had entrusted upon the first consulted physician. From the first time it was diagnosed in 1991 until 2010, BPD had been the only psychiatric illness listed in her medical file. Only very shortly before her euthanasia, the GP had been informed that she had also been diagnosed with AS. Considering that, during the whole period that she had been his patient, her severe mental crises had been linked to BPD, it therefore comes as a surprise that in the note that he wrote on the day of the euthanasia and which later would be presented by the attending physician as a first positive advice, he referred only to suffering caused by AS.^6^

Importantly, the GP seemed not to have been aware that the note indicating that he, albeit very reluctantly, supported her decision to receive euthanasia would be used as an official advice to approve her euthanasia. Instead, he believed that, in order for her to receive euthanasia, the FCECE or the Belgian Medical Association would still need to give *a priori* authorization. He certainly had no idea that the attending physician, who had unexpectedly contacted him to draw up a note and came to pick it up in a hurried visit to his cabinet, would perform the euthanasia later that evening. In his testimony, which he later repeated under oath when he appeared as a witness in the civil case against the attending physician, the GP maintained having been lured into writing what would ultimately be used as an official advice, and that had never agreed to act as a consulted physician. In its motivation of his acquittal, the Court of Assize stated that he had indeed been misled. Interestingly, this would imply that no peer control had been performed on the part of the GP, and that the euthanasia had been performed in breach of an important procedural requirement, since one of the advices necessary to make her euthanasia procedure legal had not been obtained.

### The second consulted physician (the psychiatrist)

The second consulted physician is nationally known to be among the psychiatrists generally most conducive toward euthanasia requests for unbearable mental suffering in persons with a psychiatric illness. In a 2015 study, she reported on the characteristics and outcomes of 100 euthanasia requests from psychiatric patients where she had acted as a consulted physician (26). All of these patients were diagnosed with a longstanding and treatment-resistant psychiatric illness. After the evaluation of the request, which took on average four consultations over a period of 8 months, 48 requests were granted, ultimately resulting in euthanasia on 35 patients. An analysis based on data published by the FCECE estimated that, up until 2015, the psychiatrist had acted as a consulted physician in one third to up to half of all cases of euthanasia performed on patients with a psychiatric illness in Belgium (27).

The psychiatrist had personally been approached by the patient to act as a consulted physician for her euthanasia request and had regular telephone contacts with the attending physician. Under the Law she had to act as a peer control in ascertaining both the voluntary, well-considered, and repeated nature of the euthanasia request, and the constant, unbearable, and non-alleviable mental suffering.^7^ It is rather surprising that the Law does not require the second consulted physician to also ascertain the serious and incurable nature of the illness that is causing the suffering, considering that specifically in this kind of cases that physician may well be the only person with the required expertise. Presumably, this can be explained by the fact that, when the Law was adopted, it was anticipated that only very few, if any, cases of euthanasia for mental suffering caused by psychiatric illness would ever be performed. As far as somatic illnesses are concerned, their serious and incurable nature would indeed already have been clearly established by the attending and first consulted physicians. Despite this omission from the Law, it can of course be argued that, in ascertaining whether mental suffering cannot be alleviated, it will to a large extent also be necessary to first verify the incurable nature of the illness that is causing it.

Regardless of these more general considerations, the psychiatrist acting as the second consulted physician for the euthanasia request was the only of the three physicians involved with the required competence to evaluate the nature of her illness and the characteristics of her suffering. In fact, the attending physician completely delegated the evaluation of these two crucial eligibility criteria to the psychiatrist (13). Based on her findings, the euthanasia registration document that the attending physician (belatedly) submitted to the FCECE indicated that the patient had been euthanized for unbearable suffering caused by BPD and AS. Both illnesses, it was alleged, had in her case been serious, incurable, and without prospect of improvement. However, according to our *post-hoc* analysis, the suggestions that no improvement had been possible for BPD, and that she had also suffered from AS, had not been substantiated at the time of her euthanasia.

As indicated above, the diagnosis of BPD had been established early and confirmed each time she was admitted to hospital. She had last been admitted four years before the euthanasia. Despite a long and difficult treatment history, her global functional outcome had not been poor in 2009, until a new, severe crisis emerged following the breakup of her relationship. At the end of that year, when she apparently was highly suicidal, she stopped the supportive counseling that she had been receiving from the psychologist. Afterward, the psychologist only had occasional contact with her over the phone.

The psychiatrist, in her role as second consulted physician, claimed that all treatment options for BPD had been tried and had failed. In this regard, it should be noted that, although the treatment of BPD remains a challenge and it is a long-lasting illness that cannot be “cured,” the long-term outcomes are generally better than previously expected (28–31). For the resolution of a BPD crisis, hospitalization and pharmacological interventions can be necessary. Pharmacotherapy can also be useful to treat symptoms or co-morbidities, such as depression, anxiety, and impulsivity (32–34). Outside of a crisis context, the main treatment for BPD is comprehensive psychotherapy. More specifically, recent meta-analyses and systematic reviews confirm the effectiveness of dialectic behavior therapy, schema-therapy, and mentalization-based therapy (35–44).

In 2009, these evidence-based treatments for BPD had in Belgium already been available for several years in day-hospital and ambulatory settings.^8^ It is therefore both surprising and unacceptable that the consulted psychiatrist concluded that there was no prospect of improvement for the patient’s BPD, without even having discussed and recommended these treatment alternatives. Similarly surprising is that the psychiatry expert appointed by the court also concluded that for BPD evidence-based treatments had been tried. This was substantiated by the fact that she had received a psycho-analytically oriented therapy when she was admitted to hospital at age 19, and by the assumption that she had received a behavioral therapy-based intervention at age 25, when she was admitted to a hospital where the Head was a known trainer in that approach. Regardless of whether the patient had indeed received analytic or behavioral therapy in a distant past, these statements cannot support the claim that she had been offered or had received any evidence-based treatment for BPD in recent years.

Although, according to our analysis, it had at the time of the euthanasia request not been established that her BPD was without prospect of improvement, the legal criteria would still have been fulfilled if she suffered unbearably from another serious and incurable psychiatric illness that *was* without prospect of improvement. In fact, during the evaluation of the euthanasia request, the psychiatrist referred her for an additional psychodiagnostic evaluation, resulting in the diagnosis of AS only weeks before her euthanasia. In this regard, it should be noted that in 2010 Asperger’s Syndrome or Disorder was the prototypical pervasive developmental disorder according to DSM-IV (53–56).^9^ Its core features were persistent impairments in social interaction and communication, and restrictive repetitive and stereotyped patterns of behavior and thinking, causing difficulties in social or other functioning. Later, in DSM-5, AS was removed, partly because it lacked diagnostic specificity. Instead, Autism Spectrum Disorder (ASD) was introduced as a continuum of symptoms and traits, with a specifier of severity (59–61). ASD is a usually lifelong, complex developmental disorder characterized by persistent difficulties with social communication and social interaction, and restricted, repetitive patterns of behavior causing important impairments in social or other functioning. ASD is therefore much broader than the pervasive developmental disorders in DSM-IV (62, 63).

A diagnosis of AS(D) has to be made on the basis of patient behavior, clinical symptoms, developmental history, and by conducting a psychodiagnostic assessment including specific interviews and clinical and neuropsychological testing. Screening and diagnostic tools, such as the Autism Spectrum Quotient as self-reporting instrument, are available. However, there is only limited evidence for the effectiveness of these tools in detecting AS(D) in adults with a normal range of measured intelligence, since their specificity and negative predictive value are low (64–68).

As to the euthanasia request by the patient, the psychodiagnostic evaluation that took place in March 2010 was not conducted according to the state of the art. It was not designed to offer a differential diagnosis of her symptoms, but was only performed to confirm the hypothesis of AS, by two psychologists who had been informed that the euthanasia request was pending. Moreover, although the evidence-based assessment instruments based on current screening and diagnostic guidelines had at the time already been available in Dutch, these had not been used in assessing the patient. Most importantly, the conclusion of the assessing psychologists that the patient suffered from ASD—which itself was not an official diagnosis at the time—was not justified by the results of the screening instruments that *were* used. More specifically, on five of the six tests the patient obtained normal scores, including on the putative tests for autistic traits. As evidenced by the evaluation document presented at the trial, the psychologists had merely tick-boxed the DSM-IV criteria of AS, whereas that diagnosis had not been confirmed by their test results or motivated.

Contrary to what the assessing psychologists and, consequently, the psychiatrist and the attending physician maintained, it had not been substantiated that the patient was also suffering from AS.^10^ That she was suffering from AS may actually be rather unlikely, also because such a diagnosis had never been mentioned in the numerous discharge notes of the hospitals where she had been admitted, nor been suspected by the psychologist who had for years intensively counseled her. The patient may have had some traits or symptoms that are part of the spectrum of ASD (as introduced by DSM-5). However, although there can be an overlap between symptoms of BPD and ASD, the co-occurrence of both disorders is exceptional (69, 70).

Even if the patient would have been properly diagnosed with AS, therapeutic options should have been explored before concluding that there was no prospect of improvement for that illness either. Considering that AS is a developmental disorder, no curative treatments are currently available. However, several psychosocial interventions have been developed to help patients cope with AS(D). These include: focusing on life-skills; managing challenging behavior; ensuring access to care; offering treatment for co-existing conditions; and offering family interventions (67, 71–76). Yet, after her diagnosis of AS she did not try any treatment. The psychiatrist claimed that she had suggested treatment, but that the patient had refused because there was a long waiting list and she did not expect any improvement within a reasonable timeframe. The psychiatrist also asked the patient to see a second psychiatrist, to also evaluate the euthanasia request. That person stated in writing that he could not formulate an advice, since he lacked the expertise necessary to evaluate whether the patient suffered from AS and whether all reasonable evidence-based treatment options had been tried, and since she had declined to visit him more than once.

At the trial, the court-appointed experts seemed, during their first expert testimony, to doubt that it had been sufficiently established that the diagnosis of AS was without prospect of improvement. When they in the meantime learnt from the testimony of the psychologist from the local community mental health center that she had provided the patient with regular supportive counseling, both in person and in 2010 still occasionally over the phone, they changed their opinion. During their second expert testimony, the experts argued that, since that type of support had been available to the patient, she had been sufficiently treated for AS. This is unconvincing, in light of the fact that supportive counseling by a psychologist can hardly be considered an evidence-based intervention for AS, especially when provided by a person who didn’t even know that the patient allegedly was suffering from that illness.^11^ It is therefore difficult to escape the conclusion that, even in the unlikely case that the patient had been suffering from AS, no evidence-based treatment whatsoever was tried for that condition. Consequently, to the extent that the euthanasia had been justified in reference to suffering caused by AS that lacked any prospect of improvement, this seems to have been in violation of the legal criteria.

The final, closely related evaluation which was delegated to the psychiatrist involved whether the patient, as a result of her psychiatric illness, was experiencing unbearable suffering that could not be alleviated.^12^ Although unbearable suffering is a central requirement in granting a euthanasia request, there is no generally accepted definition or operationalization of when suffering is unbearable (4, 22, 82–86). Whether a patient is suffering unbearably is up to that person to determine, and will depend on illness-related aspects, coping and personality styles, support from the patient’s environment, and other social factors. However, the Belgian Euthanasia Law also requires that the suffering cannot be alleviated, and the latter element is for the physicians to decide (4, 22, 87–97). Just like the conclusion that the psychiatric illness has no prospect of improvement, also the conclusion that the resulting suffering is non-alleviable can only be reached after all reasonable evidence-based pharmacological, psychotherapeutic, and psychosocial treatments have been tried and have failed. Reasonable treatment refers to the likelihood of obtaining success, within a realistic timeframe, and without causing intolerable side-effects (4, 22, 28, 29, 85, 86).

In this regard, it should be noted that up to 30% of psychiatric patients show limited or no response to treatment under routine treatment conditions. For most psychiatric disorders evidence-based treatment protocols and international guidelines are available that include criteria to determine treatment resistance. This determination is complex and needs to be comprehensive, by taking into account aspects of the disorder concerned, the effectiveness, availability, and accessibility of the treatment, factors related to the patient, the physicians, and the teams involved, as well as social and societal influences (4, 82, 84, 98). Table 1 gives an overview of elements that should be taken into consideration before a judgment of treatment resistance of a disorder can be made (4).

**TABLE 1:** Potential reasons for poor response and treatment-resistance (adapted from 4).

Reasons for poor response to treatment and treatment resistance
**Factors related to the disorder**
Underlying pathophysiology unknown
Multiple and interacting receptor systems
Diagnosis: categorical of dimensional
Genetic overlap between disorders
Severity of biological vulnerability
Delayed detection and treatment
Illness duration and course
Biological treatments only targeted on symptom control
**Factors related to the environment**
Severity of psychosocial stressors
History of trauma
Delayed detection and treatment
Access to EBM care/treatments
Amount of psychosocial support
**Factors related to the patient**
Severity of illness
Illness duration and course
Level of psychosocial functioning
Co-morbidities: somatic and psychiatric (including substance use/abuse)
Premorbid personality
Personal values
Coping style
Access to EBM care/treatments
Treatment adherence
**Factors related to treatment and treatment provider**
Wrong diagnosis
Wrong treatment
Lack of experience
Efficacy vs. Effectiveness
Side-effects and tolerability of treatment
Non-compliance with EBM treatment guidelines
Non-availability of EBM care/treatments

In the case under consideration the psychiatrist presumed that the patient was treatment resistant or, at least, that there was no prospect of improvement for her disorder(s) and the resulting mental suffering. The main reason for this presumption was that, at the time when the psychiatrist was contacted about the euthanasia request, the patient was mentally completely exhausted and seemingly had made up her mind that euthanasia was the only remaining solution. Following the breakup of her relationship mid-2009, her hope seemed to have vanished to ever have a successful relationship, marry, and have a family of her own. She had given up her apartment when moving in with her partner, and now had to find a new home. At the same time, her relationship with her family remained problematic, and she went on sick leave because she couldn’t cope with the additional challenge of working part-time. In anticipation of her death, she started to give away all of her belongings. Except for the physicians involved in evaluating her euthanasia request, she refused to see any other therapists, out of fear of being civilly committed and subjected to involuntary treatment. She also made it clear that she would commit suicide when her euthanasia request would be declined. Faced with this very difficult situation, the psychiatrist concluded that for the patient no reasonable options were left.

While fully appreciating the formidable challenges involved and the fact that she acted in good faith and out of a sincere feeling of compassion, the psychiatrist’s decision not to require the patient to try any evidence-based treatment cannot be accepted. The psychiatrist has repeatedly argued that, in agreeing to explore a patient’s euthanasia request, that person’s feelings of despair often disappear and new routes to treatment possibilities may open, frequently resulting in the withdrawal of the request. Although this may indeed be true, it should also be acknowledged that the exploration of alternatives to euthanasia may itself be precluded when physicians are overly receptive to euthanasia requests. In this regard, transference and counter-transference phenomena may play an important role, especially when physicians interact with patients suffering from personality disorders (2, 4, 22, 78, 79, 99, 100). Moreover, it is readily conceivable that the patient’s refusal to explore treatment options was significantly strengthened when she received the—in our opinion incorrect—diagnosis of AS, a second serious and incurable disorder. In any case, it certainly cannot be concluded that a psychiatric illness is without prospect of improvement, and that the resulting suffering cannot be alleviated, if the patient refuses to even consider treatment. The decision to nevertheless grant the euthanasia request was therefore in clear violation of the Euthanasia Law.

### *A posteriori* control by the FCECE

**FIGURE 4:**
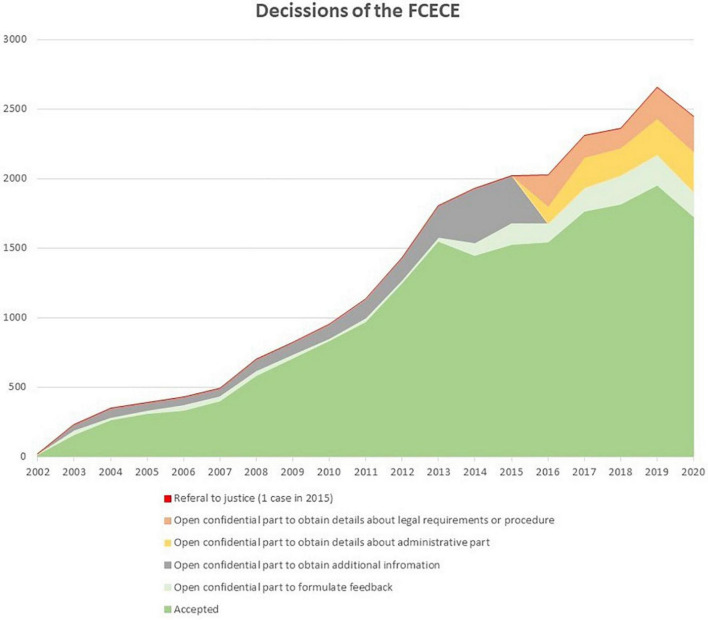
Decisions of the Federal Commission (from the official reports of the FCECE). *Category of labels changed between 2015 and 2016.

The Euthanasia Law requires that all cases of euthanasia are reported to the FCECE. This Commission performs an *a posteriori* check of each reported case as to whether the legal criteria were fulfilled. The report that needs to be submitted by the attending physician consists of an anonymous part—which includes information on: (1) the nature of the condition; (2) the nature of the suffering; (3) the reason why it could not be alleviated; (4) the elements assuring that the request was voluntary, well-considered, and repeated; and (5) the capacity of the consulted persons, and for the consulted physicians also their advice—and a part with the identifying data of the patient, the attending physicians, the consulted physicians, and the other consulted persons. If during the examination of the information contained in the anonymous part doubts arise as to whether the legal criteria were fulfilled, the Commission can, if a majority of its 16 members wishes to do so, open the non-anonymous part. This allows the Commission to request additional information from the attending physician. Of the 24,522 euthanasia cases reported until 2020, the FCECE decided to open the non-anonymous part in 21.8% of cases, so as to obtain additional information from the attending physician (16.1%) or to provide the physician with educational feedback (5.7%) (Figure 4. Decisions of the FCECE). From 2016 onward, the non-anonymous part was opened in 29.9% of reported cases, so as to obtain additional information on administrative elements (4.5%) or the legal requirements or procedure (4.1%), or to provide educational feedback (7.1%).

If, on the basis of additional information obtained from the attending physician, the Commission decides with a two-thirds majority that the legal criteria were not fulfilled, it refers the case to the Public Prosecutor, who can decide to launch a criminal investigation. Since 2002, the FCECE has referred 1 case to the Public Prosecutor. The case was ultimately not referred to the trial court by the Council Chamber because it decided to drop all charges. It was argued that it involved a case of physician-assisted suicide and that this is not covered by the Euthanasia Law and should therefore not adhere to the same due care criteria as euthanasia.

With regard to the euthanasia of the patient, one of the co-chairs of the FCECE was called as a witness before the Court of Assize. He testified that, right before the euthanasia was performed, he had been contacted by the physicians involved and, based on their information, had advised them that all legal criteria were fulfilled. When after the euthanasia her family contacted the FCECE, it was discovered that the registration document regarding her euthanasia had not yet been received. When the document finally arrived almost 2 months after the euthanasia, the patient’s euthanasia was allegedly unanimously approved by the FCECE. In his testimony the co-chair explained and emphasized the importance of the auto- and peer-control by the physicians in a euthanasia procedure. However, as indicated above, both these types of control had been problematic in this case, and this was apparently not detected at the third and final stage of control that the Law has assigned to the FCECE.

## Discussion and concluding remarks

Our analysis of the euthanasia of the patient has highlighted substantial problems in the assessment and granting of her euthanasia request within the current legal framework. As the patient was euthanized without it having been substantiated that there was no prospect of improvement for her psychiatric illness(es) nor that her suffering could not be alleviated, the requirements of the Belgian Euthanasia Law were not fulfilled.^13^ In this regard, the three-step control system enshrined in the Law and promoted by the FCECE appears to have failed at each level. Although we have shown that the patient’s euthanasia violated the law, by no means do we want to suggest that the physicians involved should have been condemned for murder by the Court of Assize, as that kind of sanction would have been completely inappropriate.

It is, however, crucial that lessons be drawn from the euthanasia of the patient to guarantee that future euthanasia requests for mental suffering based on psychiatric illness be properly evaluated and in accordance with the law. First, considering the importance of auto and peer control, it is essential that the physicians involved in the evaluation of a euthanasia request know the Euthanasia Law and conscientiously apply its criteria. In the euthanasia of the patient, the attending physician and the GP who was officially registered as the first consulted physician both testified that their knowledge of the Law was limited.

Second, to ensure that in euthanasia requests for mental suffering caused by psychiatric illness peer control can be performed, sufficient skill and experience to evaluate the nature of the illness and the suffering should be present in at least two of the physicians involved. In the euthanasia of the patient, the GPs did not have the required expertise and needed to rely on the findings of the second consulted physician. Although strictly speaking this reliance is legally allowed, it cannot be argued that peer control is performed when only one of the physicians involved is able to conduct the required evaluation. Moreover, as indicated above, it is a shortcoming of the Law to not also require the second consulted physician to evaluate the nature of the psychiatric illness, as the psychiatrist may be the only person involved who can do so.

Third, the Euthanasia Law also requires that euthanasia requests for mental suffering can validly be made only by a competent adult. Although in the case under consideration there were rightly no concerns about the possible negative impact of the psychiatric illness(es) on the patient’s decisional capacity, caution is warranted whenever doubts would arise. In this light, it is difficult to comprehend that in euthanasia requests from persons suffering from a serious psychiatric illness a formal assessment of capacity is as a rule not considered (4, 22, 25, 78, 79, 101, 102).

Fourth, the fact that a person with a serious psychiatric illness makes repeated euthanasia requests should not automatically lead to the conclusion that the decision is well-considered. This is especially the case when, as in her euthanasia request, that person had on occasion also expressed some reluctance, and a new diagnosis was given weeks before the euthanasia. Here, as in the evaluation of the fulfillment of several other legal criteria, it is essential that reasonable evidence-based treatment options are actually explored.

Fifth and possibly most crucial, before a conclusion can be reached that a psychiatric illness is untreatable or treatment-resistant, and that the resulting mental suffering cannot be alleviated, sufficient reasonable evidence-based treatments should have been tried and failed. In this regard, it is important to note that Belgian and Dutch psychiatrists have recently established a Delphi-consensus model on the diagnostic procedures and treatment criteria to be considered in euthanasia requests from persons with psychiatric illness (103). In this model it is recommended that psychiatric diagnoses need to be established in accordance with standard diagnostic criteria (e.g., DSM) and need to be independently confirmed by two psychiatrists. The clinical diagnosis should be backed by a narrative description that takes into account the systemic and contextual elements. If a patient has a long history of psychiatric illness, additional diagnostic procedures should be restricted to those that may result in new treatment options. Only when all reasonable, guidelines-based treatment options have been attempted and have failed, should MAID be considered. On the basis of the bio-psycho-social model of psychiatric disorders, this will include biological, psychological, and social treatments. Importantly, at least one recovery-oriented intervention should also have been tried and, if necessary, measures should be taken to improve the social context of the patient. However, the experts emphasized that there is a limit to the number of treatments that a patient needs to undergo before euthanasia can be considered. In assessing whether or not psychiatric suffering may be alleviated, the following factors should be considered: (1) the duration of the psychiatric illness; (2) the possibility that the patient will respond to the proposed treatment within a reasonable timeframe; (3) the possible side-effects or negative consequences of the proposed treatment; and (4) the expertise of the physicians involved and the shared nature of their decision.

In the euthanasia of the patient, the psychiatrist claimed that, at least for AS, therapeutic options had been offered but that these were refused. As a standard principle of health law, a competent patient can always refuse treatment. However, when in the evaluation of a euthanasia request potentially effective treatments are refused, it cannot be concluded that the illness is without prospect of improvement and that the suffering is non-alleviable. A euthanasia request should therefore not be granted on the basis of such a refusal. We acknowledge the difficult position that the psychiatrist found herself in when faced with a patient who refused any intervention and threatened with suicide. We also acknowledge that, taking into account the long and complicated history of her psychiatric illness, only few evidence-based treatments might still have been reasonable to attempt, and that there is a chance that her illness and suffering might eventually have proven to be treatment-resistant. However, only when treatment resistance would have been established *after* evidence-based treatments had been tried, the euthanasia would have been legitimate. Especially considering that the patient had on occasion expressed hesitance about her euthanasia wish, there might still have been possibilities to again regain some functional stability if proper assistance had been provided.

It should be noted that the psychiatrist was left alone in addressing the patient’s refusal and suicide risk, that seemed to have resulted in eventually granting her request. When the involvement of a second psychiatrist would have been required, these difficulties could have been prevented or at least been softened. If at the time of the euthanasia request a second psychiatrist was required to have been involved, it is readily conceivable that she would have been more open toward possible treatment options, knowing that these would need to be checked by not one but two experts.

In this regard, it is also important to note that in recent years several guidelines have been issued in Belgium that provide guidance on the practice of evaluating euthanasia requests of persons with psychiatric illness—some of which were drafted in response to the euthanasia of the patient (22, 85, 104–107). Despite some differences in approach, all of these guidelines agree on the necessity of complementing the legal requirements with additional safeguards. In essence, psychiatrists should not only abide by the requirements of the Euthanasia Law but also by the recommendations contained in the guideline that the Flemish Society of Psychiatry had developed on that issue (22, 85). A significant addition of that guideline is the recommendation of a two-track approach in the evaluation of a euthanasia request, which has also been endorsed in the guidelines of two important Flemish care organizations (21, 104, 106). Whereas one track should focus on the possibility of death through a comprehensive assessment of the patient’s euthanasia request, a parallel track should remain focused on life by way of exploring all remaining therapeutic and/or recovery-based possibilities. This two-track approach is founded upon two ethical values: respect for the autonomy of the patient by taking their death wish seriously, and the duty to protect life by still exploring ways to build a meaningful life. The coexistence of these two tracks implies, among others, that the patient’s psychiatrist and other physicians must remain involved throughout the whole evaluation process. Although the focus of the physicians involved will differ, each of them should pay attention to both tracks. They should also insist that the patient continues or resumes their treatment while the request for euthanasia is under evaluation.

Some of the other recommendations contained in the guideline of the Flemish Society of Psychiatry have recently been translated into deontological standards by the National Council of the Belgian Order of Physicians (108). As a result, physicians are now under a deontological obligation to comply with the following guidelines, aimed at ensuring a high standard of care. First and foremost, at least two of the three physicians involved in evaluating a psychiatric patient’s euthanasia request should be psychiatrists. The attending physician should have regular face-to-face discussions with the consulted physicians about the fulfillment of the legal criteria. These consultations must be recorded in a written report. Although euthanasia on a non-terminally ill person who suffers from a psychiatric illness can legally be performed after at least 1 month has passed counted from the date of the patient’s first request, this time-frame is considered too short for the comprehensive evaluation of the euthanasia request. Second, a patient can only be considered untreatable if all reasonable treatment options have been attempted. The physicians must be convinced that, from an objective medical-psychiatric perspective, there is no longer any reasonable treatment that can improve the patient’s condition and alleviate their suffering. Hence, a euthanasia request cannot be granted if a psychiatric patient refuses to undergo certain evidence-based treatments. Third, the attending physician should encourage the patient to involve their relatives in the euthanasia procedure, unless there are good reasons not to do so. As is clarified in the guideline of the Flemish Society of Psychiatry, involving relatives might not only contribute positively to the grieving process of the latter, but it is also important to ascertain that the euthanasia request of the patient did not come about as a result of external pressure (21). Fourth and final, as regards the evaluation of the well-considered nature of the request, it is acknowledged that a psychiatric disorder can affect the patient’s mental competence, but that this is not automatically the case. This will require a careful and comprehensive assessment by the three physicians involved (21). More specifically, the deontological recommendations set forth four criteria to determine competence: the presence of sufficient cognitive abilities (e.g., the ability to comprehend information); the capacity to combine a properly considered choice with appropriate emotions; the absence of a direct link between the euthanasia request and a symptom of the patient’s disorder; and the ability to relate the euthanasia request to important values in life.

Our analysis of the patient’s euthanasia and the resulting criminal trial supports these guidelines and deontological recommendations, as we believe that the shortcomings of the three-step control system in this case might have been avoided if these additional safeguards had been in place.

## Data Availability Statement

The original contributions presented in this study are included in the article/supplementary material, further inquiries can be directed to the corresponding author.

## Author contributions

All authors listed have made a substantial, direct, and intellectual contribution to the work, and approved it for publication.

## Conflict of Interest

The authors declare that the research was conducted in the absence of any commercial or financial relationships that could be construed as a potential conflict of interest.

## Publisher’s Note

All claims expressed in this article are solely those of the authors and do not necessarily represent those of their affiliated organizations, or those of the publisher, the editors and the reviewers. Any product that may be evaluated in this article, or claim that may be made by its manufacturer, is not guaranteed or endorsed by the publisher.

## Footnotes

1 Hageman D, Van Assche K, De Hert M. Levensbeëindiging op verzoek op basis van een psychiatrische aandoening: descriptief onderzoek Nederland en België. *Ned Tijdschr Geneeskd.* (2022) (Submitted).

2 This Commission is responsible for the examination of the completed registration forms that attending physicians need to submit after each case of euthanasia, so as to determine whether the euthanasia was performed in accordance with the legal conditions and procedure. In addition, every 2 years the Commission also needs to submit to the Parliament a statistical report on the euthanasia practice during that period, and a report in which the application of the Law is described and evaluated. Finally, although this specific role does not directly seem to fall within the scope of the tasks that it is entrusted with under the Law, the Commission issues guidance on how, in its view, some of the legal criteria should best be interpreted.

3 The Law stipulates that the second consulted physician needs to be “a psychiatrist or a specialist in the condition in question.” Considering that here the primary diagnosis is a psychiatric illness, the second consulted physician will need to be a psychiatrist.

4 De Hert M, Thys E, Catthoor K, Van den Broeck K, Matthys F, Van Steenlandt K, et al. Media coverage of a court case and end-of-life decision-making for psychiatric patients: a content analysis of Flemish newspapers and magazines. (2022). (Submitted).

5 It should be noted that, although the information brochure where the FCECE clarified the definition of “independence” as excluding a GP was published only years after the patient’s euthanasia, this exclusion criterion had been emphasized in the legal doctrine since the Euthanasia Law was first adopted (8, 10).

6 The note, written and signed by the GP on the evening of her death on 27 April 2010, read: “I physician, GP of the patient, confirm that my patient suffers from a severe psychiatric disorder (Asperger’s Syndrome), which is unbearable to my patient, [and] which can essentially not be treated nor cured. My patient therefore wants euthanasia and has confirmed it to me several times. I support her decision as a physician and close friend of my patient, but regret her decision nonetheless.”

7 It should, however, be noted that, despite the reference by the FCECE to the “peer control” of the attending physician by the consulted physicians, the attending physician is legally still allowed to perform euthanasia if the consulted physicians deem the criteria unfulfilled and have issued negative advices.

8 For the evidence-based psychotherapeutic interventions for BPD, handbooks and protocolized treatment manuals were available in 2010: dialectic behavior therapy 1993 (45), Dutch translations 1996 and 2002 (46, 47); schema therapy 2003 (48), Dutch translation 2005 (49); mentalization-based therapy 2004 and 2006 (50, 51), Dutch translation 2007 (52).

9 Recent historical research has discredited Hans Asperger because of his involvement with the Nazi-regime in Vienna (57, 58).

10 During the trial as well as in the advices of the physicians enclosed with the registration instrument sent to the FCECE, there was a constant linguistic/semantic confusion between AS and ASD. For instance, the report from the attending physician contained the oxymoronic term ‘pronounced’ AS. The impossibility of having a diagnosis of ASD in 2010 according to DSM-IV was initially acknowledged by the panel of experts appointed during the criminal investigation but, remarkably, was disregarded, or not acknowledged, by the court-appointed panel of experts who testified before the Court of Assize.

11 In this regard, it should also be noted that during the assessment of the patient’s euthanasia request none of the physicians involved had contacted the psychologist. Moreover, the consulted psychiatrist was convinced that the supportive counseling had stopped in 2007, which makes the experts’ assumption that she was right in stating that there was no prospect of improvement (since the patient had received counseling up until her final weeks) even less plausible.

12 Data on the nature of the suffering in persons who received euthanasia in Belgium is available in the FCECE reports since 2005 (Figure 3. Nature of suffering). In most patients a combination of mental and physical suffering was reported. More detailed information is available in the reports since 2016. Over recent years in on average 3.6% of the registered cases persons had received euthanasia purely for mental suffering, in 22.2% of the cases persons had been euthanized purely for physical suffering, and in 74.2% of cases a combination of both types of suffering was reported. The percentage of persons euthanized purely for mental suffering is double the percentage of the euthanized persons with psychiatric illness (1.4%). In persons with psychiatric illness who were euthanized, mood and personality disorders are the most frequent diagnoses both in Belgium and The Netherlands. However, the percentage of persons with an autism spectrum disorder who received euthanasia in Belgium is double the number reported for The Netherlands (see text footnote 1, 26, 77–81).

13 We acknowledge that our analysis of the evaluation of the patient’s euthanasia request may have some limitations. More in particular, it is not without problems to in hindsight evaluate the quality of the assessment of complex psychiatric disorders and their treatment options back in 2009–2010 (DSM-IV). It can therefore not be ruled out that our evaluation is potentially tainted by our current knowledge of the course and outcome of these disorders (DSM-5 as well as evolving treatment options over time) and our knowledge of the outcome of the criminal trial.
